# Genome-Wide Scans for Selection Signatures in Haimen Goats Reveal Candidate Genes Associated with Growth Traits

**DOI:** 10.3390/biology14010040

**Published:** 2025-01-07

**Authors:** Zhen Zhang, Jiafeng Lu, Yifei Wang, Zhipeng Liu, Dongxu Li, Kaiping Deng, Guomin Zhang, Bingru Zhao, Peihua You, Yixuan Fan, Feng Wang, Ziyu Wang

**Affiliations:** 1 Sanya Research Institute, Nanjing Agricultural University, Sanya 572025, China; 2019105032@njau.edu.cn (Z.Z.); liuzhipeng0929@163.com (Z.L.); caeet@njau.edu.cn (F.W.); 2 Jiangsu Livestock Embryo Engineering Laboratory, Nanjing Agricultural University, Nanjing 210095, China; l18904527443@163.com (J.L.); 2022205013@stu.njau.edu.cn (D.L.); t2021104@njau.edu.cn (K.D.); zhaobru@163.com (B.Z.); fanyixuan@njau.edu.cn (Y.F.); 3 College of Veterinary Medicine, Nanjing Agricultural University, Nanjing 210095, China; wangyifei0216@126.com (Y.W.); zhangguomin@njau.edu.cn (G.Z.); 4 Portal Agri-Industries Co., Ltd., Xingdian Street, Pikou District, Nanjing 210095, China; youpeihua@agriportalfeed.com

**Keywords:** whole-genome sequencing, goat, growth traits, PNLIPRP1, Indel

## Abstract

Understanding the genetic characteristics of indigenous goat breeds is vital for their conservation and breeding. Haimen goats, native to China’s Yangtze River Delta, possess distinctive traits such as white hair, moderate growth rate, high-quality meat, and small body size. However, knowledge regarding the genetic structure and germplasm characteristics of Haimen goats remains limited. In this study, we performed 20× whole-genome resequencing of 90 goats (60 Haimen goats and 30 Boer goats) to identify single-nucleotide polymorphisms (SNPs) and insertions/deletions (Indels) associated with growth traits. Here, we analyzed population genetic structure and genome-wide selection signatures between the Haimen and Boer goats based on whole-genome resequencing data. The principal component analysis (PCA) and neighbor-joining (N-J) tree results demonstrated significant genetic differentiation between the Haimen and Boer goats. The nucleotide diversity (Pi) and linkage disequilibrium (LD) decay results indicated higher genomic diversity in the Haimen goat population. Furthermore, selective sweep analysis identified candidate genes associated with growth traits. These genes exhibited strong selection signatures and were related to body size (DONSON, BMPR1B, and EPHA5), muscle development (GART, VGLL3, MYH15), and fat metabolism (ADAMTS5, LRP6, XDH, CPT1A, and GPD1). We also identified growth-related candidate genes (NCOR1, DPP6, NOTCH2, and FGGY) specific to Haimen goats. Among these genes, pancreatic lipase-related protein 1 (PNLIPRP1) emerged as the primary candidate gene influencing growth phenotypes. Further analysis revealed that a 26 bp Indel in PNLIPRP1 increased its gene expression, suggesting that this Indel could serve as a molecular marker for early marker-assisted selection, potentially enhancing early growth in goats. These findings provide valuable molecular markers and candidate genes for improving growth traits in Haimen goat breeding.

## Simple Summary

The genetic mechanisms underlying the growth phenotypic variation in the Haimen goat have not been fully revealed. This study uncovered the genetic structure and genome-wide selection signatures of Haimen goats, with a sequencing depth of 20×. Through selective sweep analysis, we identified the PNLIPRP1 gene that determines the growth traits of goats. Our study not only benefits the conservation of Haimen goats but also provides valuable insights into potential breeding strategies for this important goat breed.

## 1. Introduction

Livestock growth performance is a key economic indicator that influences national living standards. Body size (BS) in livestock is a major determinant of growth rate, energy metabolism, and body composition, all of which are regulated by multiple genes [1,2]. The identification of key genes controlling growth traits has become a central focus for researchers. In recent years, molecular markers such as single nucleotide polymorphisms (SNPs), insertion and deletion (Indel), and copy number variants (CNVs) have been widely applied in marker-assisted selection, significantly improving goat breeding programs [3,4].

With the rapid advancement of sequencing technology, whole genome sequencing has become a vital tool [5]. Utilizing whole genome sequencing, numerous studies have focused on the genetic factors that influence economic traits and production performance in goats. From the perspective of population genetics, the Fst method is more suitable for the detection of signatures of selection that have occurred in a more distant time [6]. In particular, a series of candidate genes related to domestication and artificial selection of domestic animals was determined. For example, the mutation genotype pattern located in the promoter region of HMGA2 regulates myoblast proliferation and affects body size of sheep [7]; the Indel of the L1-BT element in introns of the ASIP gene were significantly correlated with carcass and fat-related traits of Simmental steer [8]. The KIT gene related to coat color is under strong positive selection during the domestication of pigs [9].

Haimen goats, native to China’s Yangtze River Delta, have the characteristics of white hair, early maturity, and high reproduction rate, but they are small in size and poor in meat production performance. As an excellent meat goat breed in the world, the Boer goat has the advantages of having a large body, fast growth, and high slaughter rate; the Boer goats were introduced for crossbreeding to improve the productivity of native or indigenous breeds. While crossbreeding with Boer goats has been explored to improve local breeds, studies on optimizing the genetic potential of Haimen goats are still limited [10]. Selective sweep analysis was used to perform genome-wide association studies (GWAS) in Haimen goats, identifying genes associated with growth traits, including only MSTN, IGF1, MDF1, and GDF9 [11].

Identifying significantly associated SNPs/Indels with growth traits in goats would increase the efficiency of animal selection in breeding strategies by reducing the cost and time of rearing and phenotyping at weaning and fattening. Growth is a complex trait, and it is well known that it is governed by many genes, with variants having a small impact. Therefore, the ability to incorporate genetic variants into selection strategies is likely to accelerate the rate of improvement of such body weight (BW) and BS in comparison to traditional phenotypic selection. The aim of this study was to conduct a selective sweep analysis to detect possible genomic regions and variants associated with growth traits in Haimen goats, with the potential for application of the results to genomic selection.

## 2. Materials and Methods

### 2.1. Experimental Animals and Measurement of Phenotype

The experimental protocols involving goats were conducted in accordance with the guidelines provided by the Ethics Committee of Nanjing Agricultural University, China (SYXK2022-0031). A total of 60 Haimen goats from Nantong, Jiangsu Province, and 30 Boer goats from Baoji, Shanxi Province, were used. All goats were unrelated for at least three generations and housed under similar husbandry conditions. A total of 585 goats were measured at 3 months (90 ± 15 days) and 6 months (180 ± 15 days) to assess growth performance. Key parameters measured included body weight (BW/kg), body length (BL/cm), body height (BH/cm), chest circumference (CC/cm), chest depth (CD/cm), and chest width (CW/cm). Blood samples (10 mL) were collected from the wing vein using vacuum tubes containing the anticoagulant EDTA and stored at −20 °C for subsequent analysis.

### 2.2. Genome Sequencing, Variant Calling, and Annotation

Genomic DNA was extracted using a Blood Genomic DNA Extraction Kit (TRANSGEN, EE121) and adjusted to 25 ng/µL. For each sample, we constructed a paired-end sequencing library with a 300–500 bp insert size. The libraries were then performed on the DNBSEQ-T7 sequencing platform. In order to ensure the data quality, the raw data were controlled with FASTP [12] and filtered with the following criteria: (a) bases with the Phred score Q 20 in reads constitute more than 50% of the total base; (b) reads with adapter fragment; (c) reads with excessive 5 nucleotides (N); and (d) reads with a length of less than 100 bp. The clean reads were aligned to the goat reference genome (GCA_001704415.2-ARS1.1) with BWA-MEM software (version v202112.06). Variants were tested using Sentieon to obtain the gVCF files for each sample [13]. Joint analysis of gVCF across all samples yielded the variation results for each individual in the population. We then used the GATK v4.1.7 with default parameters to call SNPs. SNP and Indel sites for the 90 pooled samples were initially filtered through the following filtering parameters: QD < 2.0; FS > 60.0; MQ < 40.0; MQRankSum < −12.5; and ReadPosRankSum < −8.0 [14]. Finally, high-quality SNPs with (a) average coverage depth ≥ 5, (b) genotype detection rate above 90%, (c) MAF ≥ 0.05, and (d) dimorphism were kept for further analysis. All the filtered SNPs were functionally annotated with SnpEff [15].

### 2.3. Identification of Selection Signatures

The population differentiation index between the two goat populations was calculated using VCFtools v0.1.16, with a sliding window of 50 kb and a step size of 25 kb [16]. Empirical *p*-values were generated by genome-wide ranking of all comparisons, and the top 5% windows were considered candidate selection regions. For candidate interval genes, Gene Ontology (GO) and Kyoto Encyclopedia of Genes and Genomes (KEGG) enrichment analysis of resulting protein sequences using g: Profiler [17] and KEGG PATHWAY enrichment analysis of resulting protein sequences using KOBAS 3.0 [18].

### 2.4. Primer Design and Amplification

SNP and Indel primers were designed using the Oligo 7 software based on the DNA sequence from Ensemble. mRNA expression test primers were based on the NCBI sequence (https://www.ncbi.nlm.nih.gov/datasets/gene/taxon/9925/, accessed on 2 July 2023). Primer sequences are listed in Tables S1 and S2, respectively. Based on the BW data, DNAs from the top 15 and bottom 15 individuals were selected and pooled. A total of 4 mixing pools were used for subsequent primer validation and preliminary genotyping validation. The PCR reaction system contained 10 µL 2 × Taq Plus Master Mix II (Vazyme, Nanjing, China), 0.6 µL of internal and external primers (10 pmol/µL), 25 ng of genomic DNA (25 ng/µL), and 7.8 µL ddH2O. The procedure is as follows: 95 °C for 5 min; 95 °C for 10 s; 55 °C for 30 s; 72 °C for 60 s; the above steps for 35 cycles; and finally, at 72 °C for 7 min. The PCR products were sequenced to detect mutations.

### 2.5. SNP/Indel Verification and Correlation Analysis

SNPs were validated via the MassARRAY assay (COMPASS AGRITECHNOLOGY, Beijing, China), while Indels were analyzed using PCR and 2% agarose gel electrophoresis. Genotype and allele frequencies were calculated using Excel. Growth traits were correlated with SNPs/Indels using SAS software (V8.0). A General Linear Model (GLM), Yijk = µ + Tj + Eijk, was applied, where Yijk represents the phenotype; µ is the population mean; Tj is the genotype effect, and Eijk is the random error. Primer information used in the MassARRAY, PCR, and quantitative real-time PCR (qRT-PCR) assays is provided in Supplementary Tables S1–S3.

### 2.6. HE and ORO Staining

Liver tissue samples were fixed in 4% paraformaldehyde, dehydrated with alcohol, and cleared with xylene before being embedded in paraffin. Sections of 30 μm thickness were cut and subjected to HE staining. For ORO staining, liver tissues were prepared as frozen sections and conducted according to the protocol outlined in reference [19]. All stained sections were visualized using an inverted fluorescence microscope, and images were captured for further analysis.

### 2.7. Western Blotting Assay

Total protein was extracted from tissue samples with RIPA lysis buffer (ThermoFisher Scientific, 89901, Waltham, MA, USA) and quantified via the BCA kit (Beyotime, P0010, Haimen, Jiangsu). The sample protein was separated by SDS-PAGE (ACE, ET15420Gel) and transferred to PVDF membranes (Millipore, 88520, Burlington, MA, USA). After blocking with 5% skim milk for 2 h at room temperature, the membranes were incubated with primary antibody (PNLIPRP1 Monoclonal antibody, Proteintech (Rosemont, IL, USA), 67603-1-Ig) and secondary antibody (HRP-conjugated Goat Anti-Rabbit IgG (H + L), Proteintech, SA00001-2). After washing with TBST (Tris-buffered saline-Tween-20), the bands were visualized using ECL (Biosharp, BL520B-2, Heifei, China) and quantified with ImageJ software (version 1.42 q) (National Institutes of Health, Bethesda, MD, USA).

### 2.8. Statistical Analysis

All experiments were performed in triplicate. Data were analyzed using an independent *t*-test and one-way ANOVA with Tukey’s post-hoc test in SPSS (version 23.0). All data are presented as mean ± SEM, with *p* < 0.05 considered statistically significant.

## 3. Results

### 3.1. Sequencing and Variation Calling

**Figure 1:**
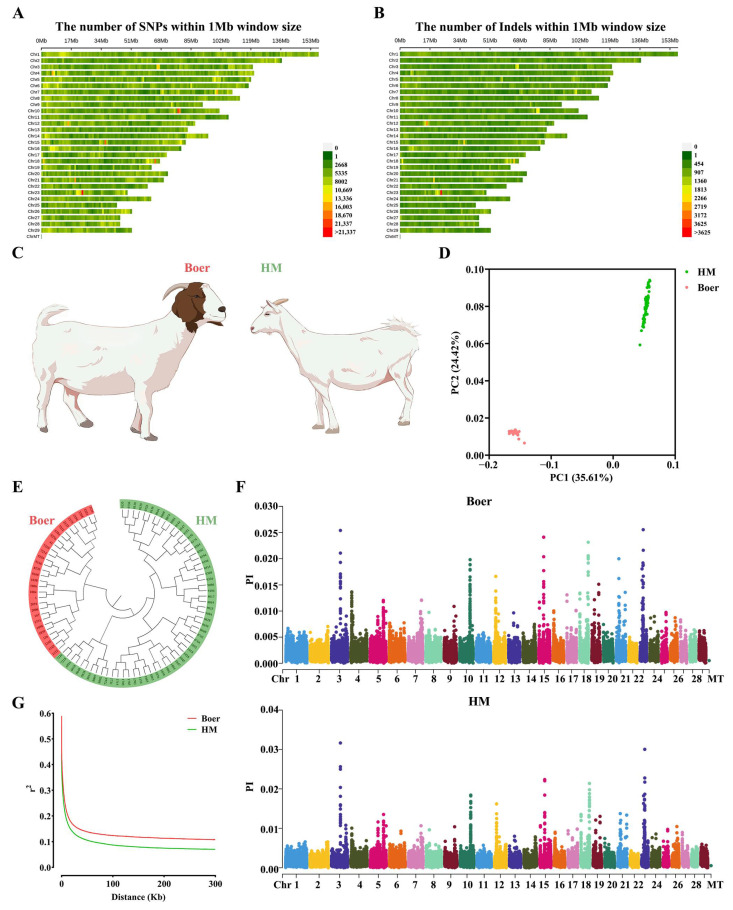
Population genetics analyses and genome-wide scanning. (**A**,**B**) The distribution of SNPs and Indels on autosomal chromosomes of goats. (**C**) Representative photograph of Boer and Haimen goat (HM). (**D**) Principal component plot. The first (PC1) and second (PC2) principal components are displayed. (**E**) Neighbor-joining phylogenetic tree of 30 Boer and 60 HM. (**F**) Distribution of nucleotide diversity (Pi) in Boer and HM. (**G**) LD decay in two species.

In this study, whole-genome resequencing was conducted on 90 individual goats, generating an average of 20× coverage per sample. The sequencing reads were aligned to the goat reference genome (Capra hircus ARS1.1), yielding 471,909,913 reads, which covered 96.89% of the reference genome (Supplementary Tables S4 and S5). Among the identified variants, 62,070 were missense SNPs, and 94,461 were synonymous SNPs (Supplementary Table S6). Additionally, 1,677,193 Indels were detected, covering 95.24% of the reference sequence (Supplementary Table S7). The majority of these variants occurred in transcripts, followed by introns and intergenic regions (Supplementary Tables S8 and S9). Whether SNP or Indel, their distribution is roughly proportional to the autosomes of goats and can be used for subsequent analysis (Figure 1A,B).

### 3.2. Population Genetic Structure

Population structure analysis based on the 90 goats revealed clear genetic differentiation between Haimen and Boer goats (Figure 1C). Principal component analysis (PCA) showed that PC1 accounted for 35.61% of the genetic variance, separating the two groups distinctly (Figure 1D). A neighbor-joining (NJ) tree further highlighted the significant divergence between Haimen and Boer goats (Figure 1E). Nucleotide diversity (Pi) analysis indicated that Haimen goats had higher genetic diversity compared to Boer goats (Figure 1F). From the perspective of linkage disequilibrium (LD), Boer goats had a slower LD decay and higher LD levels, whereas Haimen goats exhibited faster LD decay and lower LD (Figure 1G).

### 3.3. Functional Enrichment Analysis of Selected Candidate Regions

**Figure 2:**
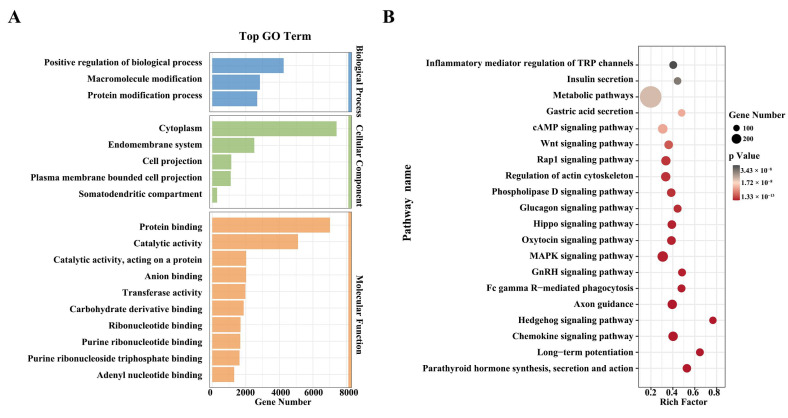
Functional enrichment analysis of candidate genes. (**A**) GO term enrichment for genes within the top 5% candidate region. (**B**) KEGG analysis of the selected genes within the candidate region.

A total of 1610 genes were identified using Fst methods (Supplementary Table S10). GO analysis revealed 25 significantly enriched biological processes, including “positive regulation of biological process”, “macromolecule modification”, “protein modification process”, “cytoplasm”, “endomembrane system”, “cell projection”, “protein binding”, “catalytic activity”, and “anion binding” (Supplementary Table S11 and Figure 2A). Additionally, KEGG pathway analysis identified 141 significantly enriched pathways (Supplementary Table S12). Among these, pathways related to production (e.g., MAPK signaling pathway, Parathyroid hormone synthesis, secretion, action, Hippo signaling pathway, Rap1 signaling pathway, Wnt signaling pathway, and calcium signaling pathway), reproduction (e.g., GnRH signaling pathway and oxytocin signaling pathway), immune response (e.g., long-term potentiation and inflammatory mediator regulation of TRP channels), and metabolic pathways such as fatty acid metabolism and glycolysis/gluconeogenesis were notably enriched (Figure 2B). After preliminary calculation of population genetic parameters and phenotype-genotype association analysis, the results showed that a total of eight variants (rs652752535, rs654084728, DPP6 g.3352780G>A, NOTCH2 g.97297615G>C, rs636776710, PNLIPRP1 26 bp Indel, and PNLIPRP1 26 bp Indel) were associated with growth traits in Haimen goats (Table 1, Tables S13 and S14). Among these variants, multiple variant sites were simultaneously located on the PNLIPRP1 gene, and therefore, PNLIPRP1 was selected as a candidate gene for further study.

### 3.4. Identification and Analysis of the PNLIPRP1 Gene

**Figure 3:**
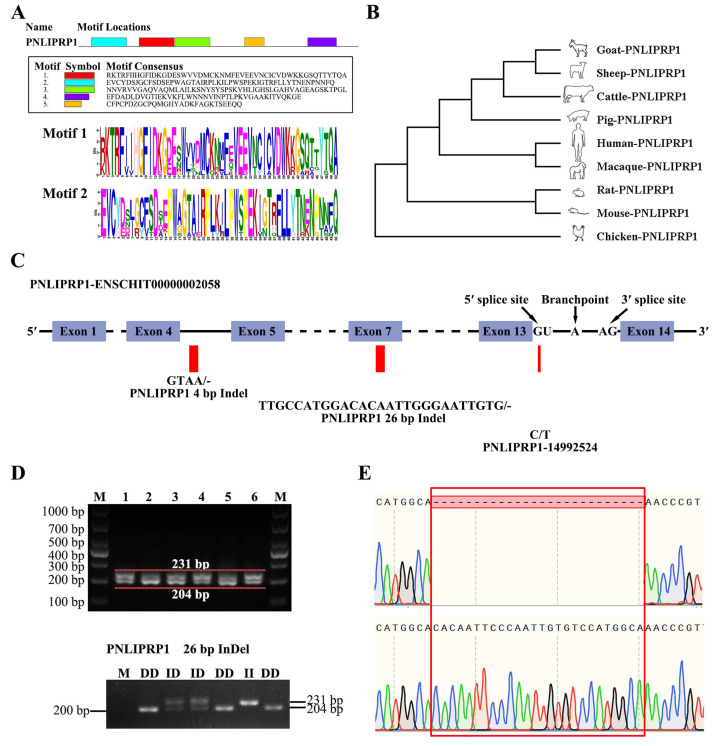
SNP and Indel analysis of the PNLIPRP1 gene in goats. (**A**) Schematically displaying Motif 1 and Motif 2 of the PNLIPRP1. (**B**) Gene evolution tree analysis of PNLIPRP1 in different species. (**C**) SNP and Indels were detected in PNLIPRP1, the loci of which are indicated in the schematic structure of the PNLIPRP1 gene. (**D**) Electrophoretic profile of amplified PNLIPRP1 26 bp Indel in 2% agarose gel. II genotype: 231 bp; ID genotype: 231 bp and 204 bp; DD genotype: 204 bp. (**E**) DNA sequencing maps of the 26 bp Indel in the PNLIPRP1 gene. The sequence with the red border shows the difference in sequence fragment.

PNLIPRP1 gene sequences and protein sequences (including goat, sheep, cattle, pig, human, macaque, mouse, rat, and chicken) were derived from the NCBI (https://www.ncbi.nlm.nih.gov/, accessed on 22 July 2024) database. The conserved protein motifs of the PNLIPRP1 gene were predicted using the online analysis tool MEME Suite 5.5.6 (https://meme-suite.org/meme/, accessed on 22 July 2024), where the parameter was set to maximum, the number of motifs was 5, and the length of motifs ranged from 6 to 50 aa (Figure 3A). The MEGA was used for multiple sequence alignment of this gene (parameter–local pair–maxiterate 1000) [20] and constructed the evolutionary tree. The analysis revealed that goats and sheep exhibited the highest genetic similarity, followed by cattle and pigs, while chickens showed the lowest level of homology (Figure 3B).

### 3.5. Effect of PNLIPRP1 Gene Polymorphism on Goat Growth Traits

The PNLIPRP1 gene was mapped to chromosome 26 in goats. The allele and corresponding genotype frequencies for the studied population of goats for various fragments of the PNLIPRP1 gene are presented in Table 1. The rs652752535 SNP was located in intron 13, identifying as a splice donor variant and intron variant (Figure 3C and Figure S1A). Three genotypes were detected for rs652752535 with the frequency of variant TT as 0.7602, TC as 0.2177, and CC as 0.0221. The respective allele frequencies for A and B alleles were 0.8690 and 0.1310, respectively. The length of PNLIPRP1 26 bp Indel target band showed in the gel of DD genotype was run into 231 bp; the length of band of ID genotype was 231 bp and 204 bp, and the II genotypic band was 204 bp (Figure 3D,E). In addition, the results showed that the frequency of the I allele at this locus was 100%, confirming the PNLIPRP1 4 bp Indel existed conservatively in goats (Supplementary Figure S1B). We further calculated gene homozygous (Ho), gene heterozygosity (He), the reciprocal of homozygotes (Ne), and polymorphism information content (PIC) as the previous methods described. The diversity parameters of PIC are 0.2017 and 0.2853, respectively. The PIC value of SNP < 0.25 indicates low genetic diversity. The PIC values of 26 bp Indel >0.25 and <0.5 indicate intermediate genetic diversity (Table 2). The 26-bp Indel was classified as a frameshift variant, and its polymorphism showed a significant association with growth traits such as BW, BL, BH, and CC at 3 months of age. In terms of growth performance, individuals with the DD genotype performed better than those with ID or II genotypes (Table 3). The genotype of rs652752535 is only significantly correlated with 3-month-old BW (Supplementary Tables S13 and S14). These findings suggest that the PNLIPRP1 gene is positively linked to growth traits in goats.

### 3.6. Impact of PNLIPRP1 on Hepatic Fat Deposition

**Figure 4:**
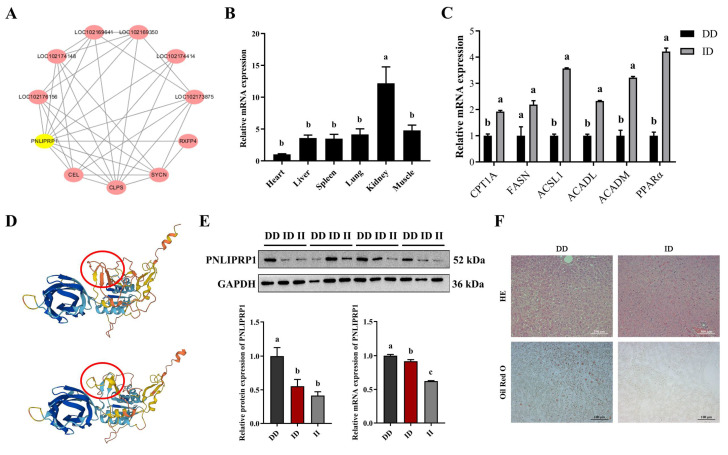
Expression pattern of PNLIPRP1 and its role in goat hepatic fat deposition. (**A**) Protein interaction network diagram constructed using Cytoscape software (version 3.7.2). (**B**) Expression of PNLIPRP1 in various tissues of 6-month-old goats. (**C**) Expression levels of fatty acid metabolism-related genes in different genotype populations. (**D**) PNLIPRP1 26 bp Indel wild-type and mutant-type protein tertiary structure prediction. (**E**) The effect of PNLIPRP1 26 bp Indel (II genotype or ID genotype) on the expression level of PNLIPRP1 in the liver. (**F**) HE and ORO staining images of liver with different genotypes; scale bars: 100 µm. Data are presented as the mean ± SEM. ^a–c^ The difference was significant when data had different letters (*p* < 0.05), whereas there was no significant difference when data had the same letters (*p* > 0.05).

Network analysis showed that PNLIPRP1 interacts with lipid metabolism-related genes (SYCN, RXFP4, CEL, and CLPS) and is enriched in the lipase activity pathway (Figure 4A and Figure S2). To preliminary explore the relationship between the Indel and PNLIPRP1 gene expression during goat development, qRT-PCR was used to find that the PNLIPRP1 gene was widely expressed in various tissues (heart, liver, spleen, lungs, kidneys, and muscle). It has been confirmed that the PNLIPRP1 gene was expressed in multiple tissues, suggesting its involvement in various developmental processes (Figure 4B). To verify this, RNA was extracted from liver tissues. It was found that the expression levels of fatty acid metabolism genes, such as carnitine palmitoyltransferase 1 (CPT1A), fatty acid synthase (FASN), acyl-coA synthetase long-chain family member (ACSL1) acyl-coA dehydrogenase long chain (ACADL), acyl-coA dehydrogenase (ACADM), and peroxisome proliferators-activated receptors (PPAR), were higher in the ID group compared to the DD group (Figure 4C). This indicates that PNLIPRP1 may promote fat breakdown and reduce fat accumulation in the liver through the upregulation of fatty acid synthesis pathways. The prediction results of the ProtParam software in Expasy (https://www.expasy.org/, accessed on 21 August 2024) showed that the molecular weight (MV) of the protein decreased from 45,197.94 g/mol to 43,230.57 g/mol before and after the mutation. The theoretical isoelectric point (Theoretical pI) changes from 8.63 to 8.66 (Figure 4D). Interestingly, we found whether homozygous or heterozygous deletions of PNLIPRP1 26 bp Indel (II genotype or ID genotype) significantly decreased the protein expression level of PNLIPRP1 in the liver (Figure 4E). Histological analysis through HE and ORO staining revealed normal liver cells in the ID genotype group, while the DD group showed signs of fatty degeneration (Figure 4F). These findings suggest that the 26-bp Indel in PNLIPRP1 could serve as a molecular marker for early marker-assisted selection in goat breeding programs, especially for growth traits and liver fat deposition.

## 4. Discussion

Identifying genomic regions linked to economic traits in livestock offers valuable insights into phenotypic variation and key genes driving these traits. It also sheds light on the mechanisms underlying artificial selection in breeding programs. In this study, we used whole-genome resequencing to identify potential variants and genes associated with growth traits in goats. Additionally, we predicted and validated how these variants impact gene structure and function.

The phylogenetic tree and PCA confirmed that Haimen and Boer goats were clearly two distinct populations, though there was evidence of gene flow between them (Figure 1D). Understanding the genetic diversity within each population can inform breeding strategies [21,22]. Nucleotide diversity and LD analysis indicated that introduced breeds had lower genetic diversity compared to Chinese indigenous breeds, likely due to more intensive breeding efforts in the introduced populations [23].

Detecting selection signatures in different populations offers fresh perspectives on phenotypic variation and helps identify genes associated with important traits [24]. Functional enrichment analysis showed that these genes were involved in pathways related to production, reproduction, and immune response, such as “Metabolic pathways”, “MAPK signaling”, and “GnRH signaling”. Metabolic pathways, which include carbohydrate metabolism, lipid metabolism, glutamine metabolism, and nucleotide metabolism, are crucial for maintaining energy balance during growth [25]. Studies have found that MAPK signaling pathways were associated with the economic traits of livestock and poultry [26,27].

Over the last decade, many genome-wide selection signatures have been detected in different domesticated goat breeds that were associated with production [28], adaptation [28,29,30], cashmere fiber [31], and multiple traits [32]. There are no such studies on the growth traits of Haimen goats except for three genes: MDFI; MSTN; and GH. We identified candidate genes linked to 3- or 6-month-old BW and body size (Supplementary Tables S13 and S14). In our study, PNLIPRP1 26 bp Indel is significantly associated with 3-month-old BW, BL, BH, and CC. Previous studies showed that the genetic variants of the PNLIPRP1 gene were associated with human carbohydrate and cholesterol metabolism disorders. Obviously, we found that the DD and ID genotypes of Indel in two goat breeds are the majority, and there are few II genotypes. The process of goat evolution began in very ancient times, accompanied by artificial selection aimed at eliminating some inferior traits and stabilizing superior ones [33,34]. Individuals with the D allele tend to have superior growth traits, indicating that selective breeding for this genotype could enhance growth performance.

Pancreatic lipase is critical for the digestion and absorption of dietary fats [35]. PNLIPRP1 was identified as being preferentially lost in herbivorous species and has no lipase activity, which is primarily associated with particular feeding habits from an evolutionary perspective [9]. Although PNLIPRP1 is lowly expressed in various tissues of goats, it is relatively differentially expressed in the kidneys. Its expression may be specific to the developmental stage, as there are reports that gene expression and various metabolic activities are active during the embryonic stage, and various metabolic enzymes are in the active stage [36,37]. It has been demonstrated that PNLIPRP1 null variants are associated with metabolic traits, such as glycemia, LDL-cholesterol levels, HDL, waist-to-hip ratio, waist circumference, and BMI [38]. Both DPP6 and FGGY genes have been associated with an increased susceptibility for sporadic amyotrophic lateral sclerosis [39]. The comprehensive QTLs/CGs analysis of 76 QTLs/CGs with RNA-seq data identified DPP6 as the locus with the highest number of SNPs in all three bovine breeds and at all developmental ages [40]. In this study, DPP6 (g.3352780G>A) is consistently strongly associated with the BL and BH of 3-month-old goats, possibly due to its involvement in processes related to skeletal muscle development. FGGY is an enzyme of the carbohydrate kinase type that uses substrates of different sugars (trioses to heptoses) to regulate processes such as the metabolic energy balance of animals [41]. In cattle, FGGY was located near the rs42518459 region, which overlapped with 12 QTLs described for several production traits [42]. We analyzed and identified the FGGY (rs641287990) genotype in 275 Haimen goats. By identifying the FGGY (rs641287990) genotype of 275 goats, the analysis showed that compared with CT genotype individuals, the BL, BH, CC, and CD of 6-month-old CC genotype goats significantly increased by 3.03 kg, 3.17 cm, 3.82 cm, and 2.04 cm. Numerous studies have demonstrated that mutations in the NOTCH2 gene were associated with various economic traits in livestock, including production traits in pigs, productive and adaptive traits in sheep, and plumage color in poultry [43,44,45]. As expected, NOTCH2 g.97297615G>C was significantly correlated with 6-month-old BL, BH, and CC (Supplementary Tables S13 and S14). The findings indicate both overlap and specificity of SNPs associated with growth traits in this study compared to other resequencing studies in goats. Therefore, it is reasonable to conclude that these SNPs in goat genes may serve as effective markers to guide molecular breeding efforts.

Frameshift mutations are assumed to have a high (disruptive) impact on protein, probably causing protein truncation, loss of function, or triggering nonsense-mediated decay [46]. Among these variants, PNLIPRP1 showed the strongest selection signal. PNLIPRP1 26 bp Indel, located in exon 7 of the gene, alters protein structure. In our study, this mutation likely disrupts the amino acid sequence, reducing protein stability. Functional studies confirmed that this deletion promotes PNLIPRP1 expression in the liver, enhancing dietary fat absorption and contributing to increased BS. In conclusion, expanding the reference population size and developing an optimal imputation strategy to achieve high accuracy in imputing more loci will enhance the identification of causal mutations in goat breeding.

## 5. Conclusions

Our study explored the genetic diversity and selection signatures of goats through selective sweep analysis, identifying growth-related candidate genes. The PNLIPRP1 26 bp Indel on chromosome 26 significantly influences BW and BS, with functional analyses suggesting its role in fat accumulation. Furthermore, several candidate genes for growth traits were detected, including NCOR1 (rs654084728), DPP6 (g.3352780G>A), NOTCH2 (g.97297615G>C), and FGGY (rs641287990). These findings provide valuable insights into the genetic makeup, population structure, and potential breeding strategies for improving goat breeds.

## Figures and Tables

**Table 1:** Candidate SNP and Indel related to Haimen goat growth trait.

Locus	Autosome	Variant	Gene	Position	Reference	Alternate Base	Type
rs652752535	26	SNP	PNLIPRP1	14992524	C	T	Splice donor variant and Intron variant
rs654084728	19	SNP	NCOR1	33085630	G	A	Missense variant
DPP6 g.3352780G>A	4	SNP	DPP6	3352780	G	A	Splice donor variant and Intron variant
NOTCH2 g.97297615G>C	3	SNP	NOTCH2	97297615	G	C	Missense variant
rs636776710	3	SNP	COL6A3	3897592	T	G	Missense variant
rs641287990	3	SNP	FGGY	34104364	C	T	Missense variant
PNLIPRP1 26 bp Indel	26	Indel	PNLIPRP1	14998429	TTGCCATGGACACAAT TGGGAATTGTG	-	Frameshift variant
PNLIPRP1 4 bp Indel	26	Indel	PNLIPRP1	15000823	GTAA	-	Splice region variant

**Table 2:** Genetic parameters of PNLIPRP1 variations in goat populations.

Locus	Genotypic Frequencies			Allelic Frequencies		Diversity Parameters				
						Ho	He	Ne	PIC	Hardy-Weinberg
rs652752535	TT	TC	CC	T	C					
	0.7602 (*n* = 447)	0.2177 (*n* = 128)	0.0221 (*n* = 13)	0.8690	0.1310	0.7724	0.2276	1.2947	0.2017	0.2905
PNLIPRP1 26 bp Indel	DD	ID	II	D	I					
	0.6034 (*n* = 353)	0.3504 (*n* = 205)	0.0462 (*n* = 27)	0.7786	0.2214	0.6553	0.3447	1.5261	0.2853	0.6892

Note: DD, homozygous deletion; II, homozygous insertion; ID, heterozygous; Ho, gene homozygous; He, gene heterozygosity; Ne, the reciprocal of homozygotes; PIC, polymorphism information content.

**Table 3:** Association analysis between different genotypes of PNLIPRP1 variations and growth performance of 3-month-old goats.

Locus	Genotype	BW (kg)	BL (cm)	BH (cm)	CC (cm)	CD (cm)	CW (cm)
rs652752535	TC	10.7 ± 0.44 ^a^	43.0 ± 0.78	44.0 ± 0.71	50.5 ± 1.03	18.9 ± 0.47	14.2 ± 0.34
	TT	9.4 ± 0.29 ^b^	42.9 ± 0.51	44.3 ± 0.46	48.9 ± 0.67	18.2 ± 0.31	13.9 ± 0.22
	CC	7.5 ± 1.42 ^b^	39.9 ± 2.51	40.9 ± 2.27	49.1 ± 3.31	18.3 ± 1.51	13.3 ± 1.10
PNLIPRP1 26 bp Indel	DD	9.4 ± 0.55 ^a^	43.8 ± 1.24 ^a^	45.9 ± 1.06 ^a^	49.1 ± 1.41 ^a^	18.1 ± 0.57	13.6 ± 0.42
	ID	8.3 ± 0.61 ^ab^	41.4 ± 1.36 ^b^	43.8 ± 1.16 ^b^	47.4 ± 1.54 ^ab^	17.7 ± 0.62	13.3 ± 0.47
	II	7.9 ± 0.98 ^b^	37.6 ± 2.21 ^b^	41.2 ± 1.89 ^b^	43.3 ± 2.51 ^b^	17.4 ± 1.01	13.1 ± 0.76

Note: Phenotypic value is shown as the mean ± SEM. ^a,b^ The difference was significant when data had different letters (*p* < 0.05), whereas there was no significant difference when data had the same letters (*p* > 0.05). BW, body weight; BL, body length; BH, body height; CC, chest circumference; CD, chest depth; CW, chest width.

## Data Availability

The data presented in this study are available upon request from the corresponding author.

## Supplementary Materials

The following supporting information can be downloaded at https://www.mdpi.com/article/10.3390/biology14010040/s1. The data used to support the findings of this study are included in this article.
