# Supplementary material for: Genome-Wide Scans for Selection Signatures in Haimen Goats Reveal Candidate Genes Associated with Growth Traits
Source: Biology (Basel). 2025 Jan 7;14(1):40. doi: 10.3390/biology14010040 (PMC11759764; doi:10.3390/biology14010040)
Supplement: Supplementary file 1 [file biology-14-00040-s001.zip › Supplementary Figure.pdf]

Supplementary information:

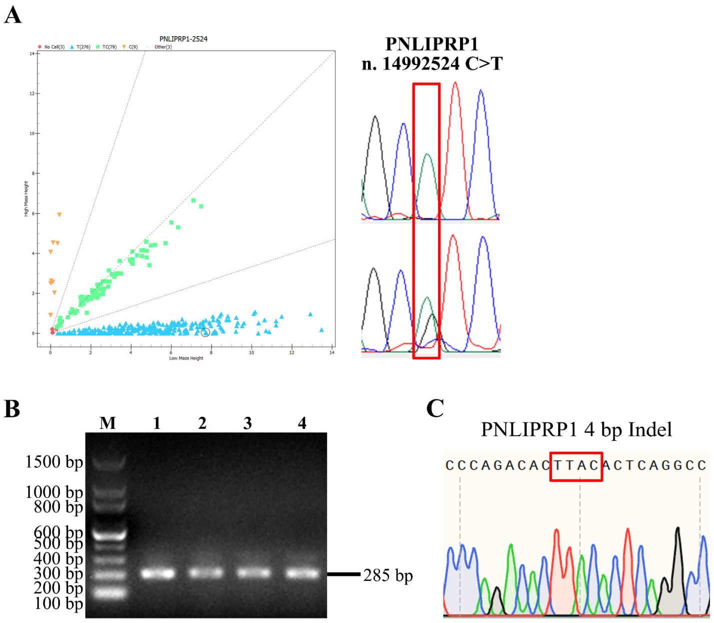

Figure S1. A: rs652752535 was validated via the MassARRAY assay and Sanger sequencing, respectively. **B:** Electrophoretic profile of amplified PNLIPRP1 4 bp Indel in 2% agarose gel. II genotype: 285 bp. **C:** Partial sequencing maps of PNLIPRP1 4 bp Indel.

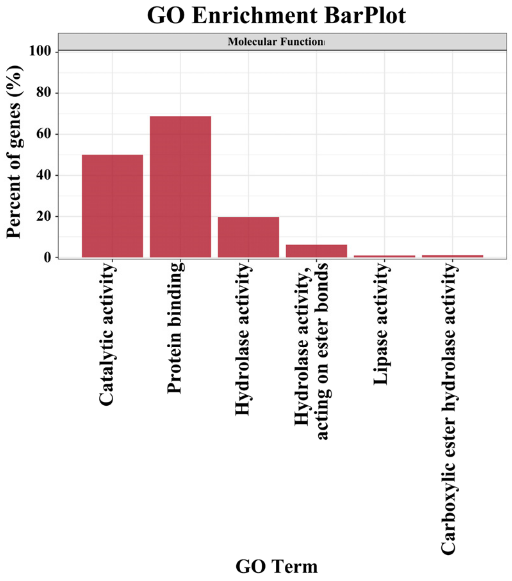

Figure S2. Enrichment of GO terms related to lipid metabolism pathways.
